# Maggot Extracts Alleviate Inflammation and Oxidative Stress in Acute Experimental Colitis via the Activation of Nrf2

**DOI:** 10.1155/2019/4703253

**Published:** 2019-11-15

**Authors:** Rong Wang, Yongzheng Luo, Yadong Lu, Daojuan Wang, Tingyu Wang, Wenyuan Pu, Yong Wang

**Affiliations:** ^1^State Key Laboratory of Analytical Chemistry for Life Science & Jiangsu Key Laboratory of Molecular Medicine, Medical School, Nanjing University, Nanjing 210093, China; ^2^School of Chemistry and Life Sciences, Nanjing University Jinling College, 210089, China; ^3^Neonatal Medical Center, Children's Hospital of Nanjing Medical University, Nanjing 210008, China

## Abstract

Ulcerative colitis (UC) is a common chronic remitting disease driven through altered immune responses with production of inflammatory cytokines. Oxidant/antioxidant balance is also suggested to be an important factor for the recurrence and progression of UC. Maggots are known as a traditional Chinese medicine also known as “wu gu chong.” NF-E2-related factor-2 (Nrf2) transcription factor regulates the oxidative stress response and also represses inflammation. The aim of this study was to investigate the effects of maggot extracts on the amelioration of inflammation and oxidative stress in a mouse model of dextran sulfate sodium- (DSS-) induced colitis and evaluate if the maggot extracts could repress inflammation and oxidative stress using RAW 264.7 macrophages stimulated by lipopolysaccharide (LPS). In the present study, we found that the maggot extracts significantly prevented the loss of body weight and shortening of colon length in UC induced by DSS. Furthermore, DSS-induced expression of proinflammatory cytokines at both mRNA and protein levels in the colon was also attenuated by the maggot extracts. In addition, the maggot extracts could significantly suppress the expression of interleukin- (IL-) 1*β*, IL-6, TNF-*α*, NF*κ*B p65, p-I*κ*B, p22-phox, and gp91-phox in LPS-stimulated RAW 264.7 cells and colonic tissues. The maggot extracts increased the level of Nrf2 and prevented the degradation of Nrf2 through downregulating the expression of Keap1, which resulted in augmented levels of HO-1, SOD, and GSH-Px and reduced levels of MPO and MDA. However, after administering an Nrf2 inhibitor (ML385) to block the Nrf2/HO-1 pathway, we failed to observe the protective effects of the maggot extracts in mice with colitis and RAW 264.7 cells. Taken together, our data for the first time confirmed that the maggot extracts ameliorated inflammation and oxidative stress in experimental colitis via modulation of the Nrf2/HO-1 pathway. This study sheds light on the possible development of an effective therapeutic strategy for inflammatory bowel diseases.

## 1. Introduction

Ulcerative colitis (UC) is one of the most common chronic inflammatory bowel diseases (IBD) which is characterized by dysfunction of the innate and adaptive immunity. UC typically demonstrates colonic mucosal injuries and histological changes in the intestines manifested by body weight loss, altered stool consistency, bloody feces, and colonic shortening [1–3]. UC mostly affects young and middle-aged people, and up to 18% of patients suffer from a chronic active disease associated with significant morbidity and loss of productivity, which generally requires lifelong treatment [3]. However, the exact mechanisms underlying the pathogenesis of UC are still unclear. It is widely believed that the disease pathology is caused by a combination of environmental factors, genetic specificity, inflammation, oxidative stress, and intestinal flora imbalance [4]. Multiple factors such as overproduction of inflammatory mediators including reactive oxygen mediators [5, 6], proinflammatory cytokines, and arachidonate metabolites and neutrophil infiltration have been implicated in the pathogenesis of colitis [7, 8].

Insects are a large, unexplored, and unexploited source that may supply potentially beneficial compounds for medication [9]. Maggots, known as a traditional Chinese medicine “wu gu chong,” are the larvae of *Lucilia sericata*. Housefly maggots have been used clinically to cure individuals suffering from ecthyma, wounds, and bacterial infection of the digestive organs since the Ming Dynasty (1368 A.D.) in China [10, 11]. When combined with other drugs, the maggot extracts demonstrate beneficial effects on coma and gastric cancer as well [10]. Maggot therapy, which has strong antimicrobial activity, is the fastest and most efficient debridement therapy in wounds with necrotic tissue and infection [12]. Recent studies have shown that maggot excretions/secretions are able to suppress multiple proinflammatory responses by neutrophil, which include degranulation, chemotaxis, respiratory burst, and integrin expression. Interestingly, maggot excretions/secretions do not seem to affect the antimicrobial activities of neutrophils [13].

Controlling inflammation is essential in preventing various diseases, such as autoimmune diseases, allergies, cancer, and metabolic syndromes [14, 15]. NF-E2-related factor-2 (Nrf2), which is the most important transcription factor for protection against oxidative stress, has been known to relieve inflammation [16, 17]. Under unstressed conditions, Nrf2 is constitutively degraded through binding to Kelch-like ECH-associated protein 1 (Keap1), an adapter protein of E3 ubiquitin ligase. In the presence of oxidative stress, Nrf2 degradation is stalled, resulting in a rapid accumulation of Nrf2. Nrf2 then translocates into the nucleus and forms a heterodimer with one of the small Maf proteins [18]. Nrf2 binds to the regulatory regions of its target genes to upregulate their transcription. An Nrf2 deficiency causes an exacerbation of inflammation in a variety of murine models, such as sepsis, pleurisy, and emphysema [19–22], as well as promoting autoimmune phenotypes in some murine strains [23, 24]. Inflammation augments oxidative stress by stimulating reactive oxygen/nitrogen species- (ROS/RNS-) generating systems, such as myeloperoxidase (MPO) and NADPH oxidase (NOX), and oxidative stress is intimately involved in the execution of inflammatory cytokines and infiltration of inflammatory cells [25, 26]. It has been recently reported that the Nrf2 inducer, dimethyl fumarate, has a beneficial effect on the treatment of multiple sclerosis [27, 28], in part based on its anti-inflammatory function. These observations indicate that Nrf2 is essential for the control of inflammation and oxidative stress.

The inflammation and oxidative stress are considered to be major risk factors in the pathogenesis of chronic diseases where the macrophages are important immune cells which regulate inflammation by producing inflammatory proteins and proinflammatory chemokines, cytokines, and reactive oxygen species (ROS) [29]. Macrophages are highly sensitive to initiators of inflammation and oxidative stress such as lipopolysaccharide (LPS) and respond by releasing mediators including TNF-*α*, lL-6, IL-1*β*, and Nrf2 [30, 31]. In our study, we observed that the maggot extracts were able to inhibit the expression of proinflammatory cytokines through upregulating Nrf2 in LPS-induced RAW 264.7 macrophages.

The aim of our study was to elucidate the mechanisms underlying the therapeutic effect of the maggot extracts in suppressing oxidative and inflammatory responses. We analyzed reactive oxygen metabolites, oxidases, and reductases as well as measured the subunits of NADPH oxidase and inflammatory cytokine expression levels in the colons of mice with DSS-induced UC and LPS-induced RAW 264.7 macrophages.

## 2. Materials and Methods

### 2.1. Maggots and Maggot Extract Preparation

Blowflies were maintained in cages under the standard condition, and their larvae were collected. Next, maggots were transferred to a large pond filled with husks of wheat seeds, milk powder, and yeast extracts. After breeding, fresh maggots were washed three times with distilled water, heat-dried, and then ground into powder followed by the addition of 2 volumes of phosphate-buffered saline (PBS). The maggot extract homogenate was made and centrifuged at 15000 r/min for 10 min. The supernatant was collected and placed in a water bath at 70°C for 30 s, followed by centrifugation at 15000 r/min for 10 min, and the supernatant was passed through a 0.22 *μ*m filter.

### 2.2. Reagents and Experimental Animals

DSS (MW: 36000–50000) was obtained from MP Biomedicals (Santa Ana, CA, USA). Monoclonal antibodies against TNF-*α*, IL-6, Nrf2, HO-1, IL-1*β*, p-I*κ*B, and NF*κ*B p65 and GAPDH were purchased from Cell Signaling Technology (Danvers, MA, USA). Monoclonal antibodies against gp91-phox, p22-phox, and Keap1 were purchased from Santa Cruz (Santa Cruz Biotechnology (Shanghai) Co., Ltd., Shanghai, China). Six- to eight-week-old female C57BL/6 mice weighing about 20 g were obtained from Model Animal Research Center of Nanjing University and maintained in a specific-pathogen-free (SPF) environment at a temperature of 22 ± 1°C, with a relative humidity of 50 ± 1% and a light/dark cycle of 12/12 h. All the mice survived the DSS-feeding experiment. Mouse care and in vivo experimental procedures were approved by the Institutional Animal Care and Use Committee of Nanjing University according to institutional animal ethics guidelines with an ethical clearance number of 2018-035210-225A.

### 2.3. Cell Culture Conditions and Drug Treatment

The RAW 264.7 murine macrophage was obtained from Keygene (Nanjing, Jiangsu, China). Cells were cultured in DMED high glucose medium (Thermo Fisher Scientific, Shanghai, China) supplemented with 10% fetal bovine serum (Hyclone, Logan, UT, USA), 100 units/ml penicillin, and 100 mg/ml streptomycin (Hyclone, Logan, UT, USA), in humidified 5% CO_2_ at 37°C. The Nrf2 inhibitor ML385 was purchased from MedChem Express (MCE Co. Ltd., Shanghai, China). Lipopolysaccharide (LPS) was purchased from Sigma (Suzhou Heyi Biotech Co. Ltd., Suzhou, China). Cells were seeded in 6-well plate at a density of 5 × 10^5^ cells/ml. Next, ML385 dissolved in DMSO (3 *μ*M) was added into the cell culture medium followed by a 2 h incubation at 37°C. Furthermore, 2 *μ*g of LPS (1 *μ*g/ml) and 200 *μ*g of the maggot extracts (4 *μ*g/*μ*l) were added to a 6-well plate followed by further incubation for 4 h at 37°C.

### 2.4. Western Blot

Cells were lysed in RIPA lysis buffer (Beyotime Biotechnology, Shanghai, China) containing 1 mM Pierce™ phosphatase inhibitor (Thermo Fisher Scientific, Shanghai, China) and 0.1% Halt™ protease inhibitor cocktail (Thermo Fisher Scientific, Shanghai, China). The mixture was centrifuged (13000 rpm, 20 min, 4°C) and the supernatants were collected. The protein concentration was determined with a BCA protein assay kit (Pierce Biotechnology, Rockford, Illinois, USA). Next, the protein extracts were mixed with a 5x SDS-PAGE sample buffer. Equal amounts of proteins (30 *μ*g) were separated by a 10% SDS-PAGE gel and then transferred to PVDF membranes (Merck Millipore, Suzhou Heyi Biotech Co. Ltd., Suzhou, China). After blocking in a 5% BSA buffer for 1.5 h, membranes were incubated with respective primary antibodies (Nrf2, Keap1, HO-1, TNF-*α*, IL-6, IL-1*β*, p-I*κ*B, NF*κ*B p65, gp91-phox, and p22-phox) at 4°C overnight. After washing with TBST three times, membranes were incubated with a secondary antibody (Biogot Technology Co. Ltd., Nanjing, Jiangsu, China) for 1.5 h at room temperature. For visualization of the bands, all membranes were incubated with the Immobilon western chemiluminescent HRP substrate (Beyotime Biotechnology, Shanghai, China) for desired durations. The relative density of the band for the protein of interest was normalized to the band for the housekeeping gene GAPDH in each group.

### 2.5. Induction and Treatment of Experimental Colitis

Mice were randomly divided into six groups: normal group (sterile water), colitis group (DSS), treatment group (DSS+maggot extracts), mesalazine group (DSS+mesalazine), maggot extract group (sterile water+maggot extracts), and Nrf2 inhibitor group (DSS+ML385+maggot extracts). Experimental colitis in C57BL/6 mice was induced by continuous administration of DSS (3% *w*/*v*) in drinking water for 7 days. For treatment, mice received the maggot extracts with a daily dose of 1 g/kg (*w*/*w*) through an intragastric cannula for 12 consecutive days starting on the first day of DSS treatment. Alternatively, mice were similarly given the positive control drug mesalazine, an aminosalicylate anti-inflammatory drug commonly used to treat inflammatory bowel diseases including ulcerative colitis, at 200 mg/kg (*w*/*w*) as a treatment regimen (*n* = 5 for each group). ML385 (30 mg/kg) pretreatment was administered intraperitoneally 1 h before administration of the maggot extracts. The behavior of mice, body weight, and stool consistency were observed and recorded on a daily basis. After 12 days, all mice were killed and their colons and serum were collected for further analysis.

### 2.6. Assessment of Body Weight, Disease Activity Index (DAI) Score, Colon Length, and Colon Histopathology

DSS-induced mouse colitis was scored as the DAI using the described criteria. In brief, severity in body weight loss, stool consistency alteration, and bleeding was scored. Weight losses of 0, 1–5%, 5–10%, 10–20%, and >20% were scored as 0, 1, 2, 3, and 4, respectively. As for stool consistency, 0 was scored for normal well-formed particles, 1 for loose stools, 2 for semiformed stools, 3 for liquid stools, and 4 for diarrhea. Bleeding was scored 0 for no blood, 1 for trace, 2 for mild hemoccult, 3 for obvious hemoccult, and 4 for gross bleeding. Next, these subscores were added and the sum was divided by 3 to obtain the DAI scores which range from 0 to 4.

Mice were killed by cervical dissociation, and colons from the caecum to the anus were cut and their lengths were measured. About 10% of the colon was fixed in 10% buffered formalin, paraffin embedded, and stained with hematoxylin and eosin (H&E) for histopathological examination. The following histological scoring system [32] was used to grade the severity of the tissue damage induced by DSS: For the percentage of damage, 0 = no tissue damage, 1 = 1–25%, 2 = 26–50%, 3 = 51–75%, and 4 = 76–100%. For the extent of tissue damage, 1 = mucosa, 2 = mucosa and submucosa, and 3 = beyond the submucosa. For the extent of crypt damage, 1 = basal 1/3 damaged, 2 = basal 2/3 damaged, 3 = only the surface epithelium is intact, and 4 = the entire crypt and epithelium are lost. For the degree of inflammation, 1 = slight, 2 = moderate, and 3 = severe. The rest of the colons were stored in PBS for western blot and quantitative real-time reverse-transcription polymerase chain reaction (RT-qPCR).

### 2.7. Measurement of SOD, MPO, MDA, and GSH-Px Activity in Colonic Tissues

Colon samples were excised and homogenized immediately at 4°C. Protein concentration was determined quantitatively with a BCA protein assay kit (Pierce Biotechnology, Rockford, Illinois, USA). SOD, MPO, MDA, and GSH-Px activity in colonic tissues was measured by chemical chromatometry using a relevant assay kit (Suzhou Heyi Biotech Co. Ltd., Suzhou, China).

### 2.8. Enzyme-Linked Immunosorbent Assay (ELISA)

Peripheral blood was collected, and the serum was separated immediately and stored at -20°C for further analysis. The concentrations of TNF-*α* and IL-6 were measured by ELISA kits according to the manufacturer's instructions (Elabscience Biotechnology, Wuhan, Hubei, China).

### 2.9. Immunofluorescence (IF) Assays

NADPH oxidase (NOX) plays an important role in oxidative stress and intestinal inflammation of UC. Although gp91-phox is the catalytic component, its partner, p22-phox, is essential for optimal activity. Accordingly, the gp91-phox and p22-phox subunits are the most important functional entities of NADPH oxidase. For immunofluorescence staining, the sections were incubated with mouse monoclonal antibodies against gp91-phox and p22-phox (1 : 200) and subsequently with FITC- or Cy3-conjugated secondary antibodies. Images were captured under a Leica DMIRE2 confocal laser scanning microscope.

### 2.10. RNA Isolation and Quantitative Real-Time PCR Assay

Total RNA was extracted using FastPure Cell/Tissue Total RNA Isolation Kit (Vazyme, Nanjing, Jiangsu, China) according to the manufacturer's instructions. Complementary DNA (cDNA) was prepared from 500 ng of total RNA according to the reverse transcription protocol using the PrimeScript™ RT Master Mix (Takara, Beijing, China). Quantitative real-time PCR analyses were performed on duplicate samples using the Applied Biosystems Power SYBR Green PCR Master Mix (Thermo Fisher Scientific, Shanghai, China). PCR amplification was performed using QuantStudio 5 (Thermo Fisher Scientific, Shanghai, China). Relative gene expression was normalized to the housekeeping gene GAPDH. Mouse mRNA primer sequences for the target genes are listed in Table 1. The relative mRNA concentrations were calculated by *E* = 2^‐*∆∆*Ct^, and the critical threshold cycle (CT) value was measured in each reaction.

### 2.11. Histological Evaluation of Potential Toxicity

Mice were sacrificed followed by blood collection. The shapes, sizes, positions, and colors of internal organs were observed and recorded. The spleen, liver, kidneys, and colon were collected and preserved in 10% neutral formalin. The tissues were then embedded in paraffin, sectioned, and stained with H&E.

### 2.12. Statistical Analysis

All data were expressed as the mean ± standard deviation (SD) from at least three independent experiments. The difference between two groups was evaluated by the Student *t*-test. ANOVA was used to compare among three or more groups. *P* values of 0.05 or less were considered statistically significant. All analysis was performed with GraphPad Prism software (San Diego, CA, USA, version 6.07).

## 3. Results

### 3.1. Maggot Extracts Ameliorate Inflammation and Oxidative Stress in LPS-Stimulated RAW 264.7 Cells via Activation of Nrf2 in a Dose-Dependent Manner

**Figure 1:**
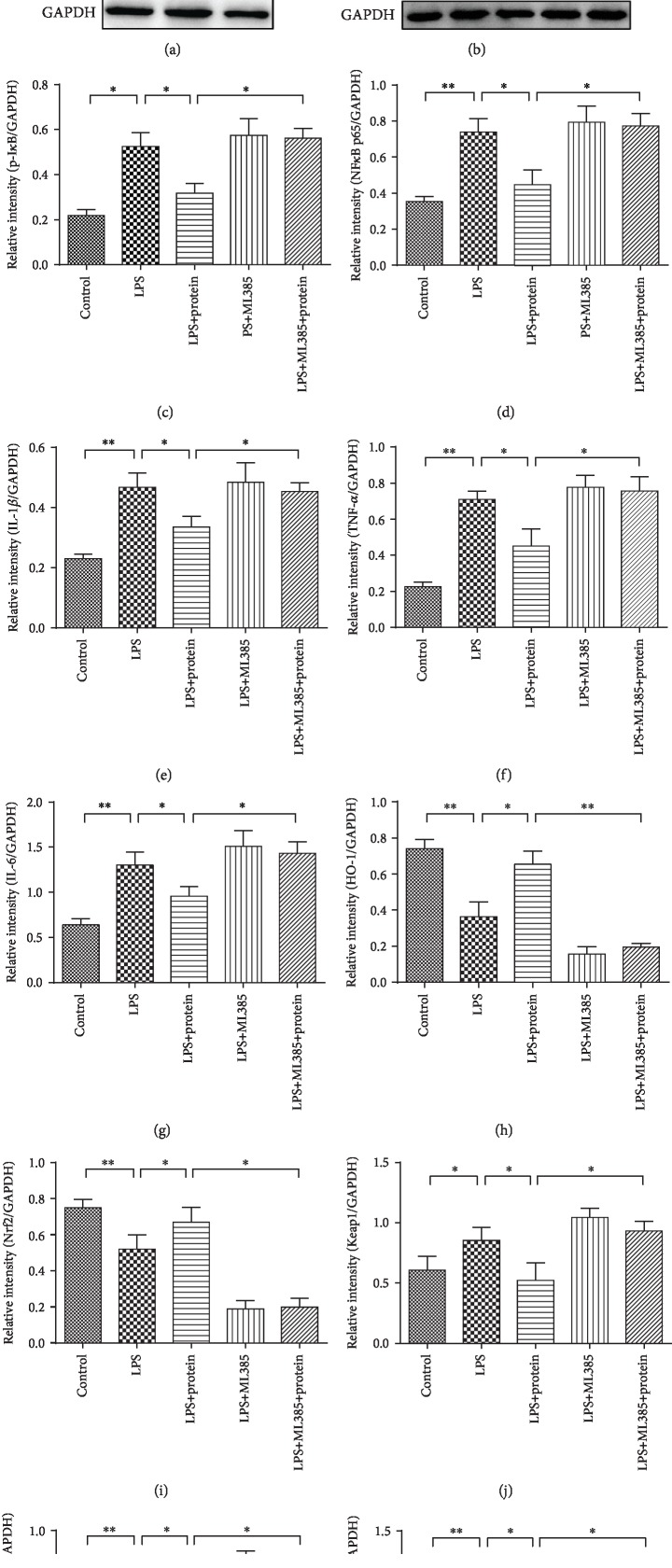
The maggot extracts are capable of suppressing inflammation and oxidative stress in LPS-stimulated RAW 264.7 cells via activation of Nrf2 in a dose-dependent manner. (a) Cells were treated with the maggot extracts for 4 h at various doses as indicated followed by analysis of the induction of Nrf2 and its downstream protein HO-1 with western blotting. GAPDH was used as an internal reference. (b) Cells were treated with a combination of LPS, the maggot extracts (4 *μ*g/*μ*l), or ML385 (3 *μ*M) as indicated followed by analysis of I*κ*B, NF*κ*B p65, IL6, IL-1*β*, TNF-*α*, Nrf2, HO-1, Keap1, p22-phox, and gp91-phox by western blotting. GAPDH was used as an internal reference. Representative data are shown. (c–l) Western blot bands shown in (b) were analyzed by densitometry. The data are shown as the mean ± SD. ^∗^*P* < 0.05, ^∗∗^*P* < 0.01, and ^∗∗∗^*P* < 0.001.

The Nrf2/HO-1 pathway regulates a battery of detoxification enzymes and antioxidant proteins including HO-1, NQO1, SOD, and GSH-Px [33]. Therefore, this pathway plays an important role in the cellular defense system. HO-1 is a rate-limiting enzyme in heme catabolism that functions as an endogenous defense mechanism [34]. Activation of Nrf2, which positively regulates the transcription of HO-1, is essential for reducing the risk of gastrointestinal inflammation [35]. An Nrf2 deficiency causes an exacerbation of inflammation in a variety of murine models, such as sepsis, pleurisy, and emphysema, which indicates that Nrf2 is essential for the control of inflammation and oxidative stress. To investigate the potential antioxidant and anti-inflammatory effects of the maggot extracts, western blot analysis was performed to detect the expression of Nrf2 and HO-1 induced by different concentrations of the maggot extracts in the LPS-stimulated RAW 264.7 cells. The results showed that maggot extracts enhanced Nrf2 and HO-1 at the protein levels in a dose-dependent manner (Figure 1(a)). Furthermore, we examined the protein expression associated with inflammation and oxidative stress in LPS-stimulated RAW 264.7 cells treated with 4 *μ*g/*μ*l maggot extracts. As shown in Figure 1(b), LPS significantly increased the expression levels of p-I*κ*B, NF*κ*B p65, IL6, IL-1*β*, TNF-*α*, Keap1, p22-phox, and gp91-phox and decreased Nrf2 and HO-1 in RAW 264.7 cells (Figures 1(c)–1(l)), indicating that LPS dramatically activated these signaling pathways associated with inflammation and oxidative stress. Interestingly, treatment with the maggot extracts markedly reversed the expression of these proteins. To further identify the underlying mechanisms accounting for the antagonizing effects of the maggot extracts in LPS-stimulated RAW 264.7 cells, we used an Nrf2 inhibitor (ML385) for the following experiments. Notably, we found that ML385 effectively inhibited the Nrf2 activity, and these effects of the maggot extracts on inflammation and oxidative stress in LPS-stimulated RAW 264.7 cells were offset by pretreatment of ML385 (Figures 1(b)–1(l)). Thus, we demonstrated here that the maggot extracts may directly inhibit upregulation of proinflammatory cytokines and oxidative stress through activation of Nrf2 and degradation of Keap1.

### 3.2. The Maggot Extracts Exert Regulatory Effects on Body Weights, Macroscopic Appearances, Colon Lengths, and the Disease Activity Index (DAI) in DSS-Induced Experimental Colitis

**Figure 2:**
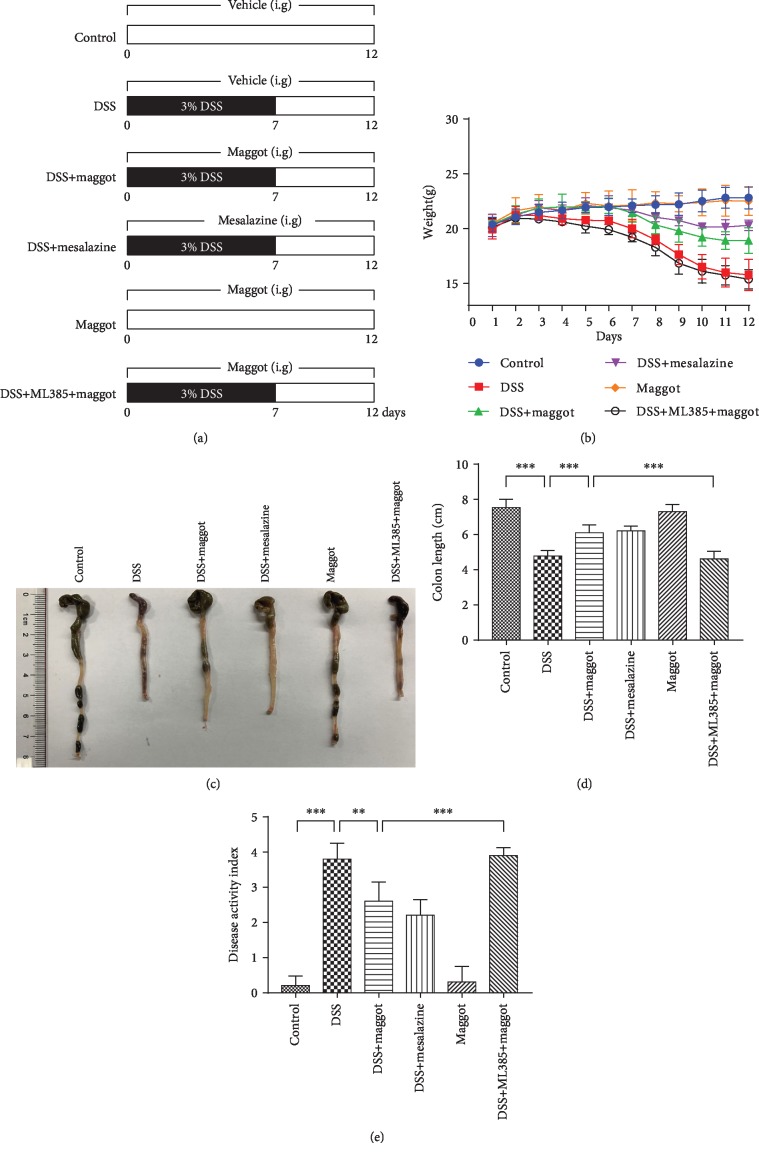
The maggot extracts prevent DSS-induced experimental colitis in mice. (a) Mice were exposed to drinking water containing 3% DSS for 7 days, followed by normal water for 5 days. Mice were given an intragastric (i.g.) administration of vehicle control, the maggot extracts (1 g/kg), mesalazine (200 mg/kg), or ML385 (30 mg/kg) from day 1 to day 12. Body weight (b), macroscopic appearances (c), colon length (d), and disease activity index (e) were measured and calculated. The data are expressed as the mean ± SD. ^∗^*P* < 0.05, ^∗∗^*P* < 0.01, and ^∗∗∗^*P* < 0.001.

To assay whether the protective effects of the maggot extracts in LPS-stimulated RAW 264.7 cells may be clinically relevant, we tested the effects of the maggot extracts on DSS-induced mouse colitis in vivo. The mouse colitis model was established as indicated in Figure 2(a). The loss of body weight, stool consistency, and bleeding are all characteristic symptoms of DSS-induced colitis [2]. We observed that loss of body weight in mice treated with DSS appeared from the fourth day. Similar to mesalazine, a commonly used drug for treating UC, the maggot extracts could robustly ameliorate the loss of body weight (Figure 2(b)). The administration of DSS induced a significant reduction in colon length, another reproducible and indirect indicator for the colitis severity of intestinal inflammation, compared with control mice (Figures 2(c) and 2(d)). The supplementation of the maggot extracts showed a significant reversal on the induction of colon shortening by DSS. Importantly, the maggot extracts and mesalazine had comparable therapeutic effects in DSS-induced mouse colitis. Compared with the mice that only received water, the DAI score, an index of the severity of colitis, was increased in DSS-exposed mice, and administration of the maggot extracts evidently improved these symptoms (Figure 2(e)). To further identify the underlying mechanisms of the maggot extracts on colitis mice, we used an Nrf2 inhibitor (ML385) for the following experiments. Based on literature reports [36, 37], we used 30 mg/kg ML385 pretreatment intraperitoneally 1 h before administration of the maggot extracts to inhibit Nrf2. Notably, we found that the protective effects of the maggot extracts on DSS-induced colitis were offset by pretreatment with ML385 (Figures 2(b)–2(e)). Thus, the results confirmed that the maggot extracts could reduce the severity of experimental colitis through activating the Nrf2 pathway.

### 3.3. The Maggot Extracts Relieve Histopathological Symptoms in DSS-Induced Experimental Colitis

**Figure 3:**
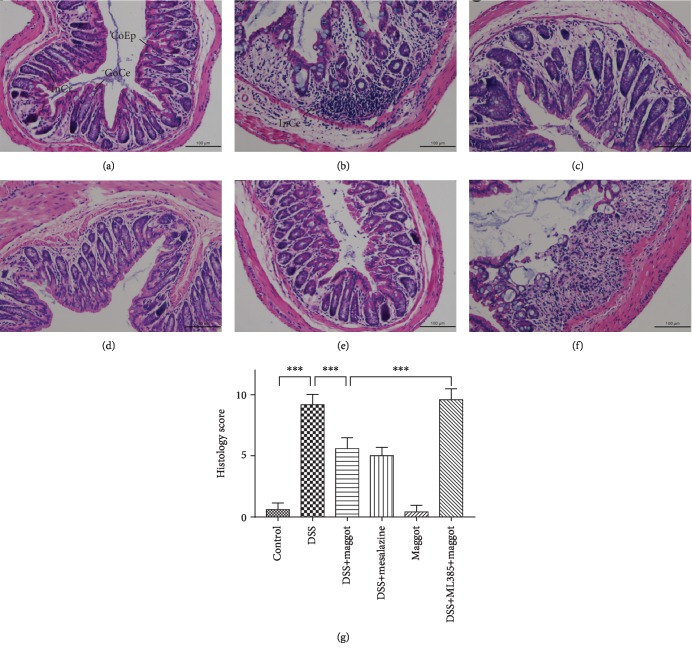
The maggot extracts alleviate DSS-induced colon damage in mice. Mice were treated as explained in Figure 2. Colons were removed and sectioned followed by H&E staining. Representative sections (a) and histology scores (g) are displayed. Scale bar = 100 *μ*m. CoEp: columnar epithelium; InCr: intestinal crypt; GoCe: goblet cell; InCe: inflammatory cell. The data are expressed as the mean ± SD. ^∗^*P* < 0.05, ^∗∗^*P* < 0.01, and ^∗∗∗^*P* < 0.001.

We further confirmed the effects of the maggot extracts on DSS-induced colitis using histopathological analyses. The acute phase of UC exhibits a number of histological features, which include mucosal erosions, crypt shortening, edema, and infiltration of inflammatory cells in the mucosa and lamina propria [38–40]. Compared with control mice (Figure 3(a)), mice treated with DSS exhibited serious injuries of colon mucosa, loss of histological structures, strong epithelial erosion, a marked decrease in the number of crypts, and pronounced infiltration of inflammatory cells (Figure 3(b)). To our surprise, mice with colitis treated with the maggot extracts exhibited reduced histological changes with respect to pathological inflammation (Figure 3(c)) comparable to mesalazine (Figure 3(d)). Administration with the maggot extracts also decreased the histological scores in DSS-induced colitis (Figure 3(g)), indicating that these extracts exerted significant protective effects on inflammation-induced intestinal damage in mice. Mice that only received the maggot extracts demonstrated no overt histological changes (Figure 3(e)). However, the colitic mice that received the maggot extracts together with ML385 pretreatment demonstrated no protective effects (Figure 3(f)). Therefore, these results indicated that the maggot extracts ameliorated histological damages in UC through activating the Nrf2 pathway.

### 3.4. The Maggot Extracts Regulate Enzymes Involved in Oxidative Stress Responses and Inflammatory Cytokines in DSS-Induced Experimental Colitis

**Figure 4:**
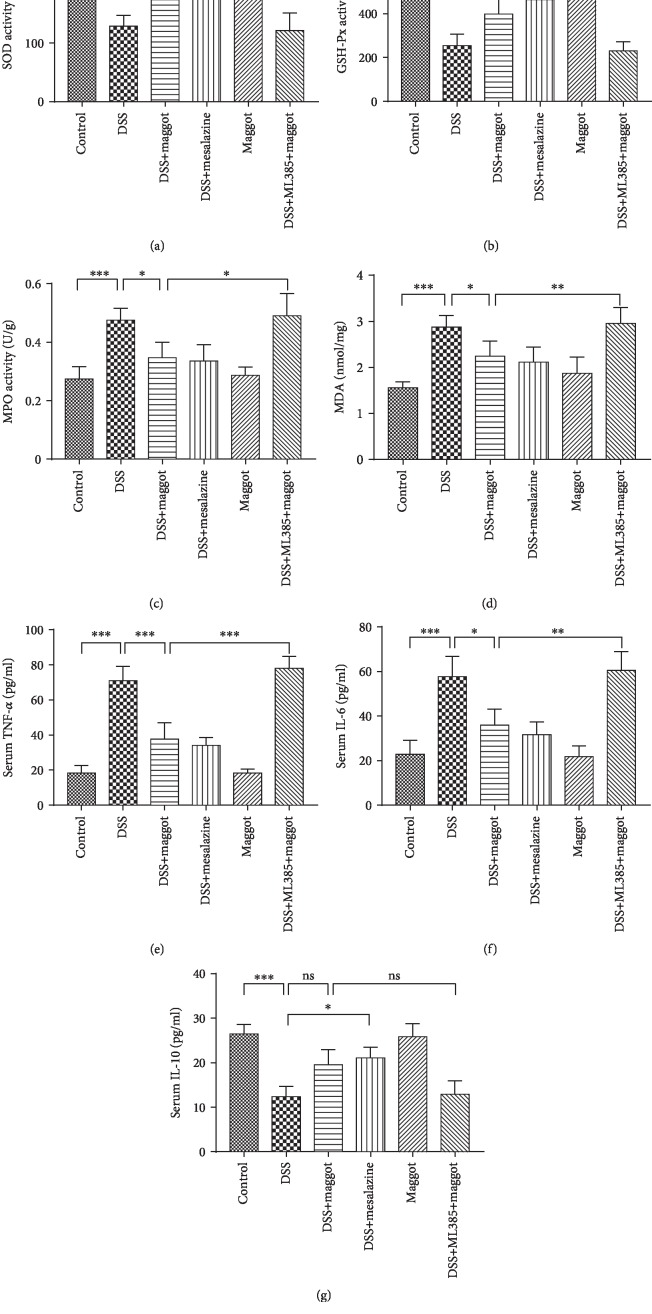
The maggot extracts reduce oxidative stress and inflammation in mice with DSS-induced colitis via activation of the Nrf2 pathway. Mice were treated as explained in Figure 2. Colonic levels of SOD (a), MPO (b), GSH-Px (c), and MDA (d) were examined by chemical chromatometry. Serum levels of proinflammatory cytokine including TNF-*α* (e), IL-6 (f), and IL-10 (g) were measured with ELISA. Data are expressed as the mean ± SD. ^∗^*P* < 0.05, ^∗∗^*P* < 0.01, and ^∗∗∗^*P* < 0.001; ns = no significance.

It is well known that ulcerative colitis is characterized by the production of a wide range of inflammatory cytokines, including TNF-*α*, IL-1*β*, and IL-6 [41]. SOD, MPO, MDA, and GSH-Px are important markers of oxidative stress in acute colitis. As shown in Figures 4(a) and 4(b), DSS induced a significant decrease in SOD and GSH-Px activities in the colon, compared with the control group. In contrast, MDA and MPO activities were higher after DSS treatment (Figures 4(c) and 4(d)). Interestingly, administration of the maggot extracts and mesalazine could markedly reverse DSS-mediated changes. Of note, the maggot extracts had no regulatory effects on naïve mice in these measurements (Figure 4). Furthermore, once ML385 was administered, the potent regulatory role of the maggot extracts in oxidative responses was suppressed (Figure 4). Next, we undertook to determine the serum levels of inflammatory cytokines by ELISA. The results demonstrated that serum levels of TNF-*α* and IL-6 in mice treated with DSS were substantially increased (Figures 4(e) and 4(f)). However, such enhancement was suppressed by maggot or mesalazine. Similarly, we also found that the protective effects of the maggot extracts on DSS-induced colitis were completely offset by pretreatment of ML385 (Figures 4(e) and 4(f)). The serum level of anti-inflammatory cytokine IL-10 was substantially downregulated in mice with DSS-induced colitis, and treatment with the maggot extracts was able to reverse the decrease, although the maggot extract-mediated suppression did not reach a statistical significance or at least demonstrated a trend of suppression (Figure 4(g)).

### 3.5. The Maggot Extracts Decrease Inflammation and Oxidative Stress via Activation of Nrf2 in DSS-Induced Experimental Colitis

**Figure 5:**
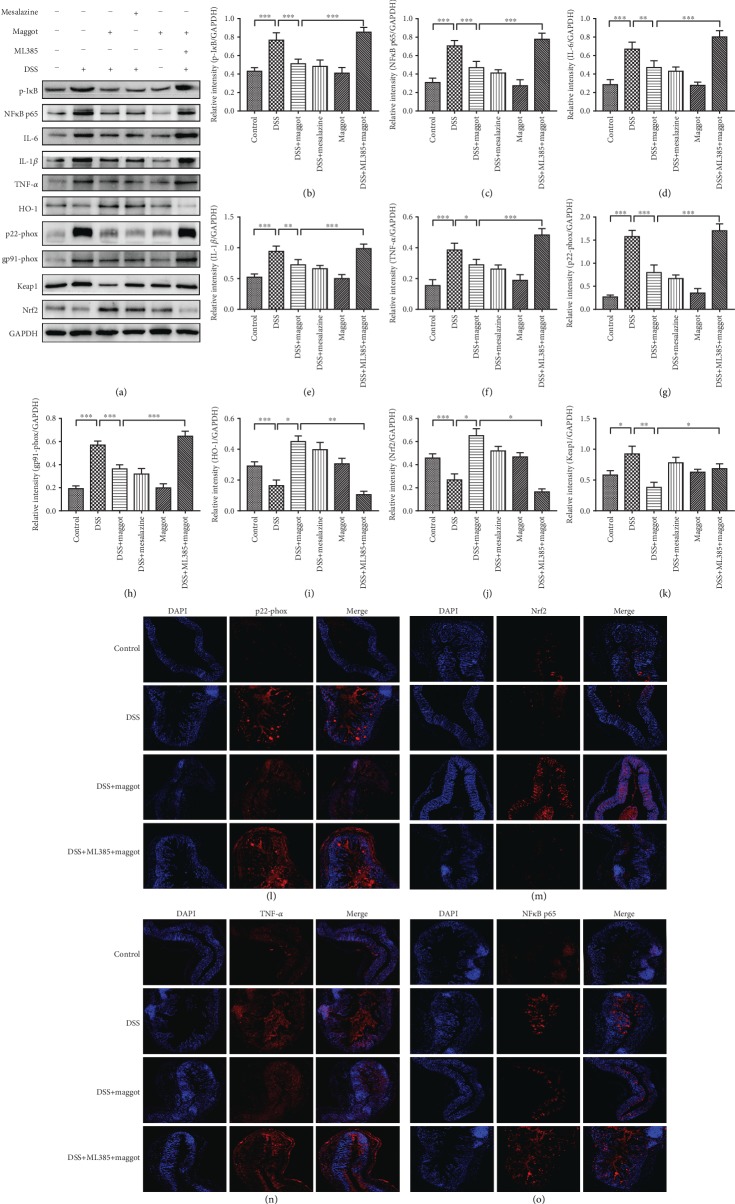
The maggot extracts exhibit anti-inflammatory and antioxidant activities in DSS-induced colitis by activating Nrf2. Mice were treated as explained in Figure 2. (a–j) The expression of p-I*κ*B, NF*κ*B p65, IL-6, IL-1*β*, TNF-*α*, p22-phox, gp91-phox, HO-1, Nrf2, and Keap1 in colonic tissues was assessed with western blotting. Representative data are displayed (a) and the relative protein intensity of p-I*κ*B, NF*κ*B p65, IL-6, IL-1*β*, TNF-*α*, p22-phox, gp91-phox, HO-1, and Nrf2 was normalized to GAPDH (b–k). (l–o) Sections of colonic tissues were immunostained for revealing various molecules (red) as indicated. The slides were counterstained with DAPI (blue), and the images were captured using an inverted fluorescence microscope. Magnification: ×200. Data are expressed as the mean ± SD (b–k). ^∗^*P* < 0.05, ^∗∗^*P* < 0.01, and ^∗∗∗^*P* < 0.001.

Our findings described above supported a potent negative regulatory role of the maggot extracts in suppressing inflammatory responses and oxidative stress. To further validate the underlying mechanism behind the protective role of the maggot extracts, we undertook to determine the signaling pathways accounting for therapeutic effects of the maggot extracts (Figure 5). As NF*κ*B plays a critical role in inflammation, we examined the effects of the maggot extracts on its activation in colon tissues. As a key regulator of detoxification, Nrf2 is responsible for the transcriptional activation of genes encoding for antioxidant enzymes. Next, we examined whether Nrf2 was responsible for the protective effects of the maggot extracts in DSS-induced colitis. Mice with DSS-induced colitis exhibited substantially increased expression of p-I*κ*B, NF*κ*B p65, IL-6, IL-1*β*, and TNF-*α* (Figures 5(a)–5(f), 5(n), and 5(o)), compared with the normal control mice. However, the expression of these proteins was markedly reversed following treatment with the maggot extracts or mesalazine (Figures 5(a)–5(f)). It has been reported that reactive oxygen species are released from activated mucosal cells during UC [42]. In order to evaluate the antioxidant activity of the maggot extracts, we examined the levels of oxidative stress-related proteins. DSS-induced colitis resulted in an increased expression of p22-phox, gp91-phox, and Keap1 in colon tissues (Figures 5(a), 5(g), 5(h), 5(k), and 5(l)). Treatment with the maggot extracts or mesalazine displayed a significant reduction of these two proteins. Furthermore, protein expression of Nrf2 and HO-1 in mice treated with DSS was significantly decreased as a result of acute colitis (Figures 5(a), 5(i), 5(j), and 5(m)). Consistently, maggot extracts and mesalazine were capable of restoring or even further enhancing the expression of Nrf2 and HO-1 (Figures 5(a), 5(i), 5(j), and 5(m)). Interestingly, the presence of the inhibitor of Nrf2 (ML385) abrogated the antagonizing effects of the maggot extracts in inflammatory responses and oxidative stress (Figure 5). Taken together, our data demonstrate that the maggot extracts exerted therapeutic effects in experimental colitis through activation of Nrf2 and degradation of Keap1.

### 3.6. The Maggot Extracts Regulate the Transcript Expression Levels of Proinflammatory Cytokines via Activation of *Nrf2* in DSS-Induced Experimental Colitis

**Figure 6:**
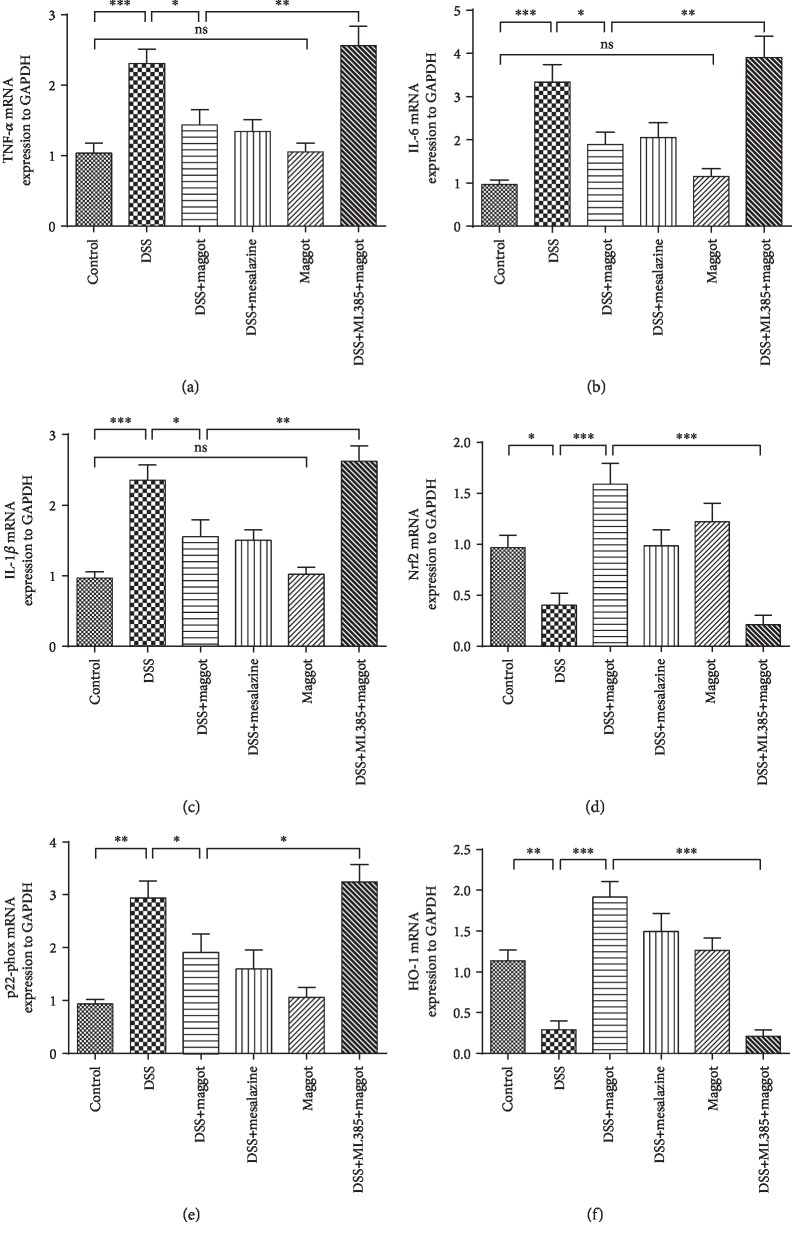
The maggot extracts regulate the proinflammatory cytokines and *Nrf2* in the colon of mice with DSS-induced colitis. Mice were treated as explained in Figure 2. mRNA expression levels of the proinflammatory genes including *TNF-α* (a), *IL-6* (b), and *IL-1β* (c) as well as oxidative stress-related proteins including *Nrf2* (d), *p22-phox* (e), and *Ho-1* (f) in colonic tissues were determined by real-time PCR. The housekeeping gene *GAPDH* was used as a loading control. Data are expressed as the mean ± SD. ^∗^*P* < 0.05, ^∗∗^*P* < 0.01, and ^∗∗∗^*P* < 0.001; ns = no significance.

It is well known that UC is characterized by the production of a wide range of inflammatory cytokines [41]. Oxidative stress and inflammation are always associated with each other, and the use of antioxidants can be protective against inflammatory diseases [43]. To determine the effects of the maggot extracts on DSS-induced colitis, mRNA levels of the proinflammatory cytokines and *Nrf2* in the colons were measured using quantitative real-time PCR. Consistent with protein expression, DSS treatment significantly upregulated proinflammatory cytokines, including *TNF-α*, *IL-6*, and *IL-1β* at the mRNA level (Figures 6(a)–6(c)). Treatment with the maggot extracts almost completely suppressed the upregulation (Figures 6(a)–6(c)). In addition, *Nrf2* and *Ho-1* at the mRNA level were significantly lower in the colons of DSS-induced mice than in healthy controls (Figures 6(d) and 6(f)). In contrast, *p22-phox* at the mRNA level was markedly upregulated in colitic mice (Figure 6(e)). Again, mice treated with either the maggot extracts or mesalazine significantly antagonized DSS-induced effects (Figures 6(d)–6(f)). However, in the presence of ML385, activation of *Nrf2* was inhibited and the protective effects of the maggot extracts on mice with DSS-induced colitis were abolished (Figure 6). These mRNA regulation data also support that the maggot extracts exerted its beneficial effects on DSS-induced colitis through the Nrf2 signaling pathway.

### 3.7. Chronic Administration of the Maggot Extracts Does Not Induce Toxicity in Major Organs in DSS-Induced Experimental Colitis

**Figure 7:**
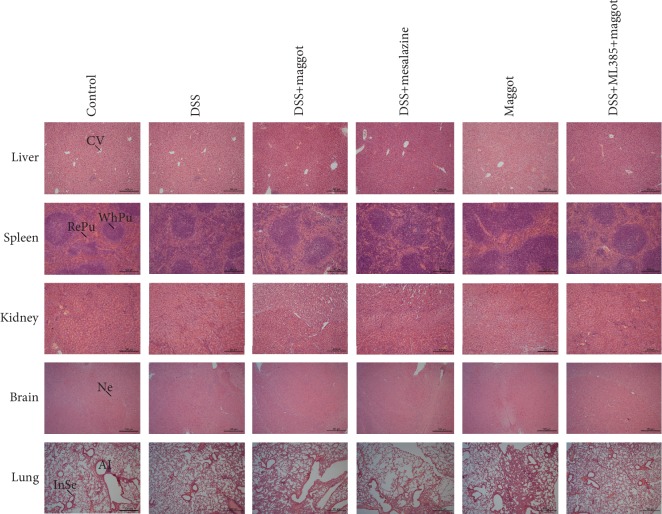
The maggot extracts demonstrate no toxicity in mice with DSS-induced chronic relapsing colitis. Mice were treated as explained in Figure 2. The spleen, liver, kidneys, brain, and lungs were sectioned and stained with hematoxylin and eosin (H&E). Scale bar = 500 *μ*m. RePu: red pulp; WhPu: white pulp; Al: alveolus; InSe: interalveolar septum; CV: central vein; Ne: neuron.

The potential toxicity of the maggot extracts after chronic administration in DSS mice was next evaluated. There were no apparent clinical signs of toxicity in any of the mice treated with these extracts. Upon microscopic examination of selected organs of mice, mononuclear cells were found to be infiltrated in the spleen after a 12-day treatment with DSS, consistent with spleen enlargement (Figure 7). However, no discernible treatment-related abnormalities were observed following the maggot extract treatment (Figure 7). Histopathological examinations of the remaining organs of DSS mice revealed that there were no patterns of clinically important abnormalities in the liver, kidney, brain, or lung (Figure 7). Accordingly, in mice treated for 12 days with the maggot extracts, no gross abnormality was seen with respect to the morphological features (Figure 7).

## 4. Discussion

UC is a recurrent and prolonged inflammatory disease common in the digestive system that is detrimental to the physical and mental health of affected patients and can possibly increase the risk of colorectal cancer [44]. The drugs that are currently in use to ameliorate the clinical symptoms and inflammation associated with UC include corticosteroids, aminosalicylates, immunosuppressors, and other biological agents. However, these drugs also exert adverse effects. Therefore, studies seeking to develop safer, effective, and targeted therapies for UC are necessary. Recent research has demonstrated that a cecropin-like peptide extracted from maggot had strong antimicrobial activities against Gram-negative bacteria and in immunomodulation [45]. Therefore, we intended to explore the protective effects of the maggot extracts on DSS-induced UC. Our data clearly demonstrated that the maggot extracts alleviated inflammation and oxidative stress in experimental colitis via modulation of the Nrf2/HO-1 pathway. While contributions of the maggot extracts to anti-inflammation have been recognized in our previous study [46], the molecular basis accounting for the function of the maggot extracts has not been elucidated. In this study, two important points have been clarified. First, we discovered that the maggot extracts could inhibit inflammation and oxidative stress response in LPS-induced RAW 264.7 macrophages and a murine model of DSS-induced colitis. Second, the maggot extracts worked by upregulating Nrf2, which further activated the expression of downstream antioxidant enzymes and repressed the NF*κ*B signaling pathway.

In our study, we first observed the dynamic changes of p-I*κ*B, NF*κ*B p65, IL6, IL-1*β*, TNF-*α*, p22-phox, gp91-phox, Keap1, and Nrf2/HO-1 in LPS-stimulated RAW 264.7 cells after administration of the maggot extracts. Furthermore, we confirmed that ML385 was able to inhibit nuclear translocation of Nrf2 in colonic tissues. Although direct interaction of Nrf2 with these inflammatory transcription factors has not been reported, MafK, one of the obligatory partner molecules of Nrf2, is reported to associate with NF*κ*B p65 [47]. Consistent with this finding, a recent report has shown that NF*κ*B p65 resides in the Nrf2-based transcription factor complex in LPS-treated Raw 264.7 cells [31]. Based on the results from our cellular assays, we further designed a mouse colitis model to verify the protective effects of the maggot extracts. DSS-induced colitis is one of the well-established mouse models that shares many signs and symptoms with human UC, including body weight loss, diarrhea, emaciation, melena, mucosal ulcerations, shortening of colon length, and inflammatory cell infiltration [48]. Body weight loss, severe diarrhea, bloody stools, and decreases in activity and appetite appeared on the fourth day after the administration of DSS. After 12 days, mice with DSS-induced colitis exhibited a remarkably higher DAI score than the control mice. Furthermore, we observed DSS-induced pathological changes in the colonic tissue samples by HE staining and found that the mice in the DSS group displayed prominent abnormalities in the colonic architecture, such as mucosal ulcerations, crypt losses, glandular defects, and inflammatory cell infiltration. Moreover, the histological scores were significantly higher after DSS treatment. Thus, 3% DSS dissolved in drinking water successfully induced UC in C57BL/6 mice. Our data confirmed that the maggot extracts alleviated the symptoms and pathological changes in the intestine that are characteristic of DSS-induced experimental UC in mice. Although the underlying causal mechanism of UC still remains unknown, our study further validated the protective effects of the maggot extracts on oxidative stress and inflammation. Oxidants play a direct role in the chronic inflammatory process by increasing the number of neutrophils and macrophages that induce a self-sustaining phlogogenic loop [49]. A conformational change in Keap1 triggered by the coupling of electrophiles to the protein results in a weakening of the Nrf2-Keap1 interaction. This causes stabilisation of Nrf2, and newly formed protein accumulates in the nucleus where it binds to antioxidant response elements in the promoter of Nrf2 target genes. Our data showed that the maggot extracts may directly inhibit upregulation of proinflammatory cytokines and oxidative stress through degradation of Keap1 and activation of Nrf2. Activation of the Nrf2/HO-1 pathway could provide an endogenous defense system to resist cellular oxidative stress and mitigate oxidative damages, making it a promising therapeutic mechanism for suppressing inflammation [50, 51]. The activation of Nrf2 downstream cytoprotective proteins, such as SOD, HO-1, and GSH-Px, establishes an endogenous defense system against oxidative stress. It is essential to prevent the intestinal epithelium cells from physical and biochemical stimulation. In addition, activation of Nrf2 reduces cell damage and relieves pathological inflammatory responses. All these protective effects demonstrate that the activation of Nrf2 can balance the cytochemical microenvironment, thereby activating a series of signaling pathways against inflammation. Recent research has revealed aggravation of acute DSS colitis in response to constitutive Nrf2 expression [18]. According to our data, we speculate that the constitutive activation and inducible expression could yield different results. NF*κ*B, a redox-sensitive transcription factor important for inflammation, innate immunity, and maintaining tissue integrity, regulates the expression of several proinflammatory factors, such as IL-6, IL-1*β*, TNF-*α*, COX-2, and chemokines. These cytokines finally induce local inflammation and immune dysfunction, changes that trigger a positive feedback loop leading to the development of inflammation, resulting in intestinal mucosal damages [52]. Those inflammatory cytokines are markedly upregulated not only in experimental colitis [53] but also in patients with UC [54]. Our results showed that SOD and GSH-Px were increased notably and MPO and MDA were reduced substantially in the colonic tissues of the DSS-induced mice. Administration of the maggot extracts or mesalazine could suppress the alteration. Furthermore, once the inhibitor of Nrf2 (ML385) was administered, the potent regulatory role of the maggot extracts in oxidative stress responses was abrogated. This clearly demonstrated that the maggot extracts effectively activated the Nrf2 pathway. Consistently, we observed an elevation of these cytokines, including TNF-*α*, IL-1*β*, and IL-6, p-I*κ*B, NF*κ*B p65, p22-phox, gp91-phox, and Keap1 in colitis mice. Interestingly, the presence of ML385 abrogated the therapeutic effects of the maggot extracts in inflammatory responses and oxidative stress. Therefore, we confirmed that the maggot extracts ameliorated inflammation and oxidative stress in experimental colitis by activating Nrf2. Importantly, no apparent clinical signs of toxicity were detected in five selected organs in addition to the intestine (i.e., lungs, kidneys, spleen, liver, and brain).

As this has been the first time we explored the therapeutic efficacy of the maggot extracts in a mouse colitis model, certain limitations existed in our study. As the DSS model induces a severe form of colitis, we started the treatment from the beginning when DSS was given. Future studies may address the therapeutic effects of the maggot extracts on an already established disease. As our crude extracts are not suitable for intravenous or intradermal injection, the maggot extracts were delivered intragastrically to mice in the current study. We have not been able to compare the effect of different delivery routes on the efficacy of the treatment. Future experiments are warranted for evaluating the therapeutic potential of this protein in its purified, injectable form. Although general compounds such as amino acids, proteins, and nutrients are not expected to activate Nrf2, some minor compounds (maybe antimicrobial peptides [45, 55]) present in the maggot extracts can contribute to the Nrf2 activation. We may try to identify the causative agents in the maggot extracts in future studies.

In conclusion, our present study demonstrated that the maggot extracts could ameliorate inflammation and oxidative stress in experimental colitis via the activation of Nrf2, resulting in an overall improvement in both macroscopic and histological parameters. Our results also demonstrated that the maggot extracts regulated the Nrf2/HO-1 and NF*κ*B signaling pathways in DSS-induced UC mice. However, the direct correlation of NF*κ*B inhibition with Nrf2 activation is yet to be confirmed. Further investigation may be designed to explore the protective effects of the maggot extracts using Nrf2-knockout and NF*κ*B-knockout mice. Our study strongly provides a potential therapeutic strategy for treating UC by exploiting the antioxidant and anti-inflammatory effects of the maggot extracts.

## Figures and Tables

**Table 1:** Sequences of primers designed for RT-qPCR.

Gene	Forward sequence	Reverse sequence
*TNF-α*	TCTCCAGCCACCAGCCCTCTAA	TGGCCATGGTAGGAGAAACAGG
*IL-6*	ACAAAGCCAGAGTCCTTCAGAG	GGCAGAGGGGTTGACTT
*IL-1β*	TCCAGGATGAGGACATGAGCA	GAACGTCACACACCAGCAGGT
*Nrf2*	AACAACGCCCTAAAGCA	TGGATTCACATAGGAGC
*Ho-1*	CACGTATACCCGCTACCT	CCAGTTTCATTCGAGCA
*p22-phox*	GCTCATCTGTCTGCTGGAGTA	ACGACCTCATCTGTAACTGGA
*GAPDH*	AGGTCGTGTGAGGGATTG	TTAGTAGTAGTAGGGGGGGTCA

## Data Availability

The data used to support the findings of this study are included within the article and all data are available.

## Abbreviations

UC: Ulcerative colitis
Nrf2: NF-E2-related factor-2
DSS: Dextran sulfate sodium
LPS: Lipopolysaccharide
IL: Interleukin
TNF: Tumor necrosis factor
SOD: Superoxide dismutase
MPO: Myeloperoxidase
MDA: Malondialdehyde
GSH-Px: Glutathione peroxidase
HO: Heme oxygenase
NF*κ*B: Nuclear factor *κ*B
IBD: Inflammatory bowel diseases
ELISA: Enzyme-linked immunosorbent assay
PBS: Phosphate-buffered saline
DAI: Disease activity index
IF: Immunofluorescence.
