# Corrigendum to “Maggot Extracts Alleviate Inflammation and Oxidative Stress in Acute Experimental Colitis via the Activation of Nrf2”

**DOI:** 10.1155/2020/3814012

**Published:** 2020-08-25

**Authors:** Rong Wang, Yongzheng Luo, Yadong Lu, Daojuan Wang, Tingyu Wang, Wenyuan Pu, Yong Wang

**Affiliations:** ^1^State Key Laboratory of Analytical Chemistry for Life Science & Jiangsu Key Laboratory of Molecular Medicine, Medical School, Nanjing University, Nanjing 210093, China; ^2^School of Chemistry and Life Sciences, Nanjing University Jinling College, 210089, China; ^3^Neonatal Medical Center, Children's Hospital of Nanjing Medical University, Nanjing 210008, China

In the article titled “Maggot Extracts Alleviate Inflammation and Oxidative Stress in Acute Experimental Colitis via the Activation of Nrf2” [1], there were errors in Figures 1 and 2. In Figures 1(c), 1(d) and 1(i), PS should be corrected to LPS. In Figure 2, the DSS group image for the liver was incorrect and should be corrected as follows.

## Figures and Tables

**Figure 1:**
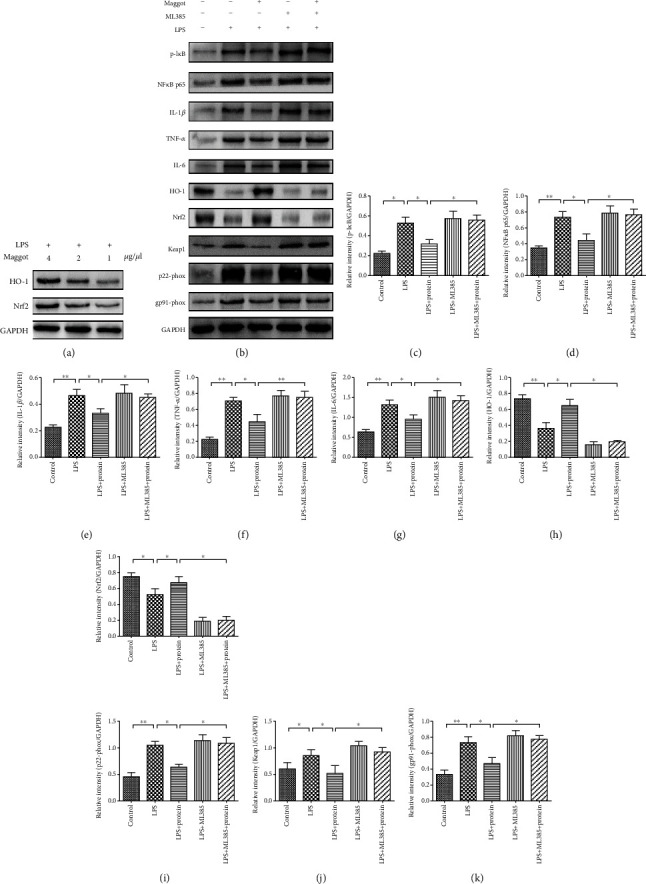


**Figure 2:**
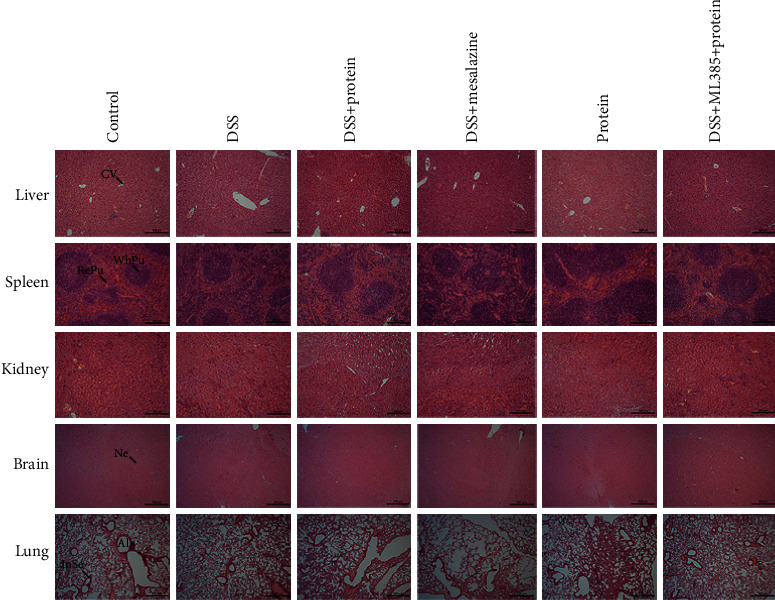


## References

[1] Wang R., Luo Y., Lu Y. (2019). Maggot extracts alleviate inflammation and oxidative stress in acute experimental colitis via the activation of Nrf2. *Oxidative Medicine and Cellular Longevity*.

